# The integrative analysis of transcriptome and metabolome reveals differences in raw *foie gras* performance induced by different force-feeding intensities in male Tianfu meat geese

**DOI:** 10.3389/fvets.2025.1653733

**Published:** 2025-09-25

**Authors:** Yongqiang Teng, Rongxue Wei, Shanjing Peng, Jiang Li, Rong Ning, Zhaoyun Luo, Shouhai Wei, Lili Bai, Li Zhou, Chunchun Han

**Affiliations:** ^1^Key Laboratory of Livestock and Poultry Multi-omics, Ministry of Agriculture and Rural Affairs, College of Animal Science and Technology, Sichuan Agricultural University, Chengdu, China; ^2^Farm Animal Germplasm Resources and Biotech Breeding Key Laboratory of Sichuan Province, College of Animal Science and Technology, Sichuan Agricultural University, Chengdu, China; ^3^Yibin Academy of Agricultural Sciences, Yibin, China

**Keywords:** goose fatty liver, transcriptome, amino acid metabolome, fatty acids metabolome, force-feeding intensity

## Abstract

We aimed to explore the influence of different force-feeding intensities on *foie gras* performance. Geese were force-fed with A (force-feeding four times per day and lasting 28 days) and B (force-feeding 5 times per day and lasting 18 days) at two levels of force-feeding intensities in this study. An integrative analysis of the liver transcriptome, amino acid metabolome, and long-chain fatty acid metabolome was performed. In serum, the levels of blood glucose, insulin, triglyceride (TG), and very low-density lipoprotein (VLDL) of the B group were significantly higher than those of the A group (*p* < 0.05). The B force-feeding intensity induced more severe steatosis in the goose liver. Transcriptome analysis showed that 948 upregulated differentially expressed genes (DEGs) and 519 downregulated DEGs were identified (A *vs.* B); key DEGs *G6PD*, *IGF1*, *IGF2*, and *MLX* were upregulated; *LPL*, *IRS1*, *IRS4*, and *IGF1R* were downregulated. Principal component analysis (PCA) indicated that there was clear separation and discrimination in liver free amino acids profiles and long-chain fatty acids profiles between the A and B groups, respectively. The Lys level of the B group was significantly higher than that of the A group (*p* < 0.05). The highest enrichment pathway of different amino acids was valine, leucine, and isoleucine biosynthesis, whereas alanine, aspartate, and glutamate metabolism was the pathway involved with the highest impact score. The levels of saturated fatty acids (SFAs), monounsaturated fatty acids (MUFAs), unsaturated fatty acids (UFAs), C14:0, C16:1, C16:0, C18:2n6c, C18:1n9c, and C18:0 of the B group were significantly higher than those of the A group (*p* < 0.05). The highest impact score pathway related to different fatty acids was linoleic acid metabolism, and the highest enrichment pathway involved in different fatty acids was biosynthesis of unsaturated fatty acids. In conclusion, liver DEGs involved in glucolipid metabolism, different free amino acids, and different fatty acids collectively shaped the *foie gras* performance difference induced by different force-feeding intensities.

## Introduction

In order to study avian growth, development, metabolism, and reproductive performance, and other problems, researchers often feed birds with different feeding methods or patterns to develop a good model or methodology system for academic experiments. In contrast to *ad libitum* feeding, there are three common feeding methods: starvation, restricted feeding, and force-feeding (1–3). In practical production, these three methods are also used to control growth or reproductive performance, for example, starvation was used for breeding hens forced molting (4); restricted feeding was used to control body fat deposition of broilers and improve reproductive performance of breeding poultry (5, 6); long-term force-feeding was used to *foie gras* production, and short-term force-feeding was used to Beijing roast duck fattening (7). The main purpose of force-feeding was to increase body fat deposition (e.g., roast duck) and liver lipid accumulation (e.g., *foie gras*) in waterfowl production. Previous studies had investigated the influence of feed consumption levels on growth performance, serum biochemical parameters, body fat deposition, liver histology, and apparent digestibility of nutrients in Pekin ducks to determine the optimum amount of feed consumption that maximized the growth performance during the force-feeding period in roast duck production (8–10). *Foie gras*, the fatty liver of force-fed ducks or geese, is the primary and most valuable product of waterfowl production. However, the feed conversion ratio was very low during ducks or geese force-feeding. In order to avoid excess feed consumption and minimize the accumulation of wastes in *foie gras* production, the optimum amount of feed consumption in *foie gras* production of mule ducks was determined (11, 12). However, the influence of different force-feeding intensities on *foie gras* performance has been seldom reported.

Force-fed geese or ducks’ livers increased in weight and size by 5 to 10 times after overfeeding with a high-energy diet, which was rich in carbohydrates, in 2 weeks. This process was accompanied by severe hepatic steatosis. The distinctive genetic characteristic of waterfowl was exploited in *foie gras* production (13). However, many studies have shown that when ducks and geese were force-fed excessive feed, nutrient digestibility, absorption, and utilization will inevitably decrease (14). When excessive carbohydrate enters the digestive tract of force-fed ducks or geese, the ducks or geese cannot fully digest the feed and directly discharge it, which can easily cause feed waste and environmental pollution. In addition, *foie gras* production is extremely controversial because of animal welfare. Thus, how to reduce force-feeding intensity or shorten the force-feeding period becomes an urgent problem to be solved (15). Therefore, in view of these problems, such as feed resource waste, environmental pollution, and animal welfare, it is of great significance to investigate the effects of different force-feeding intensities on *foie gras* performance. To preliminarily compare the influence of different force-feeding intensities on *foie gras* performance, ganders were force-fed with A (force-feeding four times per day and lasting 28 days) and B (force-feeding five times per day and lasting 18 days) at two levels of force-feeding intensities in this current study. In addition, the goose liver transcriptome, amino acids metabolome, and long-chain metabolome were conjointly analyzed, which will provide a new insight into the *foie gras* performance difference caused by different force-feeding intensities. Not only will this study provide a scientific basis for increasing feed utilization, but it will also provide an approach to improving the efficiency of *foie gras* production. Meanwhile, it will provide not only an idea for decreasing feed cost and environmental pollution, but also a reference to animal welfare.

## Methods and materials

### Birds and experiment design and sampling

After body weight selection (3,500 ± 100 g), a total of eighty 100-day-old male Tianfu Meat Geese were randomly and evenly divided into A group and B group based on A (force-feeding four times per day and lasting 28 days) and B (force-feeding five times per day and lasting 18 days) two levels of force-feeding intensities, respectively. The geese were force-fed the boiled maize (maize boiled for 5 min, supplemented with 1% plant oil and 1% salt), and allowed *ad libitum* access to water. After force-feeding started, the force-feeding intake gradually reached 160–200 g (per meal) on the 5th day. The temperature was controlled at 25 °C, and the humidity was maintained at 65 to 70% during force-feeding. When the force-fed geese became lethargic and unable to walk, they were slaughtered in time to avoid high mortality and get better *foie gras* performance. The rest of the force-fed geese were slaughtered on the 18th and 28th days, individually. After suffering from 12 h of fasting, the force-fed geese were weighed, and blood sampling (10 mL of blood was collected from the wing vein) was conducted. After slaughter, the liver tissues were collected and weighed. Each *foie gras* sample was divided into three parts: the first part was frozen in liquid nitrogen immediately, and kept at −80 °C for transcriptome sequencing, free amino acids detection and long-chain fatty acids determination; the second was kept at −20 °C for detections of dry matters, crude fat and crude protein; the third part was washed in ice-cold saline (0.9% NaCl; 4 °C) and fixed in 4% formaldehyde-phosphate buffer for histomorphology examination.

### Biochemical index examinations of serum

Ten blood samples were randomly selected from each group for the quantification of serum biochemical indices. The collected blood was centrifuged for serum separation (4,000 r/min for 10 min, 4 °C), and the separated serum was stored at −20 °C for follow-up serum parameters determination. The assay kits were provided by Nanjing Jiancheng Bioengineering Institute (Nanjing, China). According to manufacturer instructions, blood glucose, insulin, triglyceride (TG), total cholesterol (T-CHO), high-density lipoprotein (HDL), and very low-density lipoprotein (VHDL) were detected.

### Hepatic steatosis histological examination

The cross-sections from the middle of the liver were preserved in 4% formaldehyde-phosphate buffer and prepared using standard paraffin embedding techniques. Tissue sectioned at 5 μm was stained with hematoxylin and eosin (HE), sealed with neutral resin, and examined using a microscope photography system (Olympus, Tokyo, Japan) (five visual fields were randomly selected at 40 × magnification). According to the manufacturer instructions, Oil Red O (Sigma, United States) staining of liver tissue was performed in following order: the formalin-fixed liver tissue was dehydrated by 10, 20, and 30% sucrose solution in sequence; dehydrated liver tissue was embedded with OCT, and then sliced (10–15 μm) at −20 °C; the section was slightly washed with 50% ethanol after drying; after staining (the washed section was stained with oil red O solution for 8–10 min, the stained section was washed with tap water and then re-stained with hematoxylin violet), the stained section was sealed by glycerin gelatin. Liver tissue slices, which were stained with HE and oil red O, were examined by a microscope photography system (Olympus, Tokyo, Japan).

### Determinations of water, crude fat, and crude protein

The water content of liver samples was determined by the freeze-drying method with a vacuum freeze dryer (Thermo Fisher Scientific, United States). Crude fat was determined using the Soxhlet extraction method. Crude protein was measured using the Kjeldahl method. Each sample was performed in triplicate. The detailed methods involved in freeze-drying, Soxhlet extraction, and Kjeldahl determination were strictly implemented in accordance with our previous research (16).

### Liver transcriptome sequencing

A total amount of 1 μg RNA, which was isolated from a liver sample, was used as input material for the RNA sample preparations following the manufacturer’s instructions. Sequencing libraries were generated using NEBNext® UltraTM RNA Library Prep Kit for Illumina® (NEB, United States), and index codes were added to attribute sequences to each sample. The RNA samples were reverse-transcribed to cDNA using the PrimeScript™ RT reagent kit with gDNA Eraser (Takara, Japan). The library fragments were purified using the AMPure XP system (Beckman Coulter, Beverly, United States), and the cDNA fragments of preferentially 240 bp in length were selected. Then, 3 μL USER Enzyme (NEB, USA) was used with size-selected, adaptor-ligated cDNA at 37 °C for 15 min, followed by 5 min at 95 °C before PCR. PCR was performed with Phusion High-Fidelity DNA polymerase, Universal PCR primers, and an Index (X) Primer. Finally, PCR products were purified with the AMPure XP system (Beckman Coulter, Beverly, United States), and library quality was assessed on the Agilent Bioanalyzer 2,100 system. The clustering of the index-coded samples was performed on a cBot Cluster Generation System using TruSeq PE Cluster Kit v4-cBot-HS (Illumina, United States) according to the manufacturer’s instructions. After cluster generation, the library preparations were sequenced on the Illumina platform, and paired-end reads were generated. The sequencing was conducted by Baimike Biological Technology Co., Ltd. (Beijing, China).

Raw reads in fastq format were first processed through an in-house Perl script to remove adapter sequences and reads containing poly-N and of low quality. Reference genomes were downloaded directly from the NCBI genome database, and paired-end clean reads were aligned to the reference genome using HISAT2 (17). The DEseq R package was used to identify differentially expressed genes (DEGs) between these two groups (A *vs.* B). The resulting *p*-values were adjusted using Benjamini and Hochberg’s approach for controlling the false discovery rate. DEGs were identified based on the criteria of |log2FoldChange| > 2 and an adjusted *p*-value of < 0.1. GO function of DEGs was analyzed through online DAVID (v2021) (18).^1^ In addition, KOBAS (v3.0) software^2^ was used to predict the significantly enriched KEGG pathways (19).

### Determination of liver free amino acids

After grinding, 0.5 g of a fresh liver sample was measured accurately and mixed with 2 mL of 0.01 mol/L HCl in a special tube, which was specifically used for homogenization. After homogenization, the liver tissue homogenate was transferred to a 15-mL centrifugal tube. After centrifugation (8,000 × g, 10 min), the upper solution was transferred to a 10-mL volumetric flask. The settling matter was mixed with 2 mL of 0.01 mol/L HCl and oscillated by vortex oscillator for 2 min, then centrifuged again, and the supernatant was removed into the 10-mL volumetric flask. This process was repeated in triplicate. And then, diluted the supernatant with pure water to volume, and shook (sample solution preparation was finished). Six hundred μL of sample solution and 400 μL of 10% salicylic acid were accurately measured and mixed in a 2-mL centrifuge tube, and kept at 2–8 °C for 60 min (precipitated protein). After centrifugation (12,000 × g, 15 min), 500 μL of the upper solution was transferred to another 2-mL centrifuge tube for vacuum drying. After vacuum drying, the dry matter was dissolved in 1 mL of 0.01 mol/L HCl. The solution was filtered through a 0.22-μm filter membrane into the sample bottle for amino acids determination. Reversed phase high performance liquid chromatography (HPLC) (Agilent 1,260 Infinity LC, Agilent Technologies, United States) was used to detect amino acids. Ortho-phthalaldehyde (OPA) and 9-Fluorenylmethyl Chloroformate (FMOC) were the online derivatization reagents. Chromatograph conditions, automated online derivatization program, gradient elution program, and calculation of amino acids concentration were rigorously performed according to our previous study (16). The area normalization method was used to calculate the relative contents of liver free amino acids.

### Determination of liver long-chain fatty acids

An internal standard method was used to determine the long-chain fatty acids in *foie gras*. Undecylenic acid methyl ester (CAS#: 13552–80-2) (ANPEL, Shanghai, China) was used for the internal standard. *Foie gras* sample pretreatments were strictly implemented following our previous research (20). Gas chromatograph-mass spectrometry (GC–MS) (890A-5975C, Agilent Technologies, United States) was used to separate and analyze the fatty acid methyl esters. Chromatographic conditions included an HP-5MS capillary column (30 m × 0.25 mm × 0.25 μm) (Agilent Technologies, USA). The temperature program started with an inlet temperature of 220 °C, then held at 60 °C for 2 min, increased to 280 °C at a rate of 6 °C/min, and held for 10 min. The carrier gas was helium (He) with a total flow rate of 1 mL/min and a split ratio of 10:1. Mass spectrometry conditions included an electron energy of 70 eV, ion source temperature of 230 °C, a mass scan range of 50–450 u and a delay time of 3.5 min. Signals were collected in full scan mode. Detailed information on the method for calculating fatty acid concentration is provided in the Supplementary materials - Long-chain fatty acids detection.

### Data analysis

T-test and data visualization were performed using GraphPad Prism software (GraphPad 8.0 Software, Inc.) (The data will exhibit the same normal distribution). Values were means ± SD. *p* < 0.05 was considered a significant difference. Principal component analysis (PCA) was performed by R, with the “ggbiplot” package (21). MetaboAnalyst 6.0^3^ was applied to visualize enrichment analysis and the metabolic pathways involved in amino acids and fatty acids. The KEGG database was used to search for the related KEGG pathways of the fatty acids and amino acids for integrative analysis.

## Results

### Influence of different force-feeding intensities on *foie gras* performance

**Figure 1:**
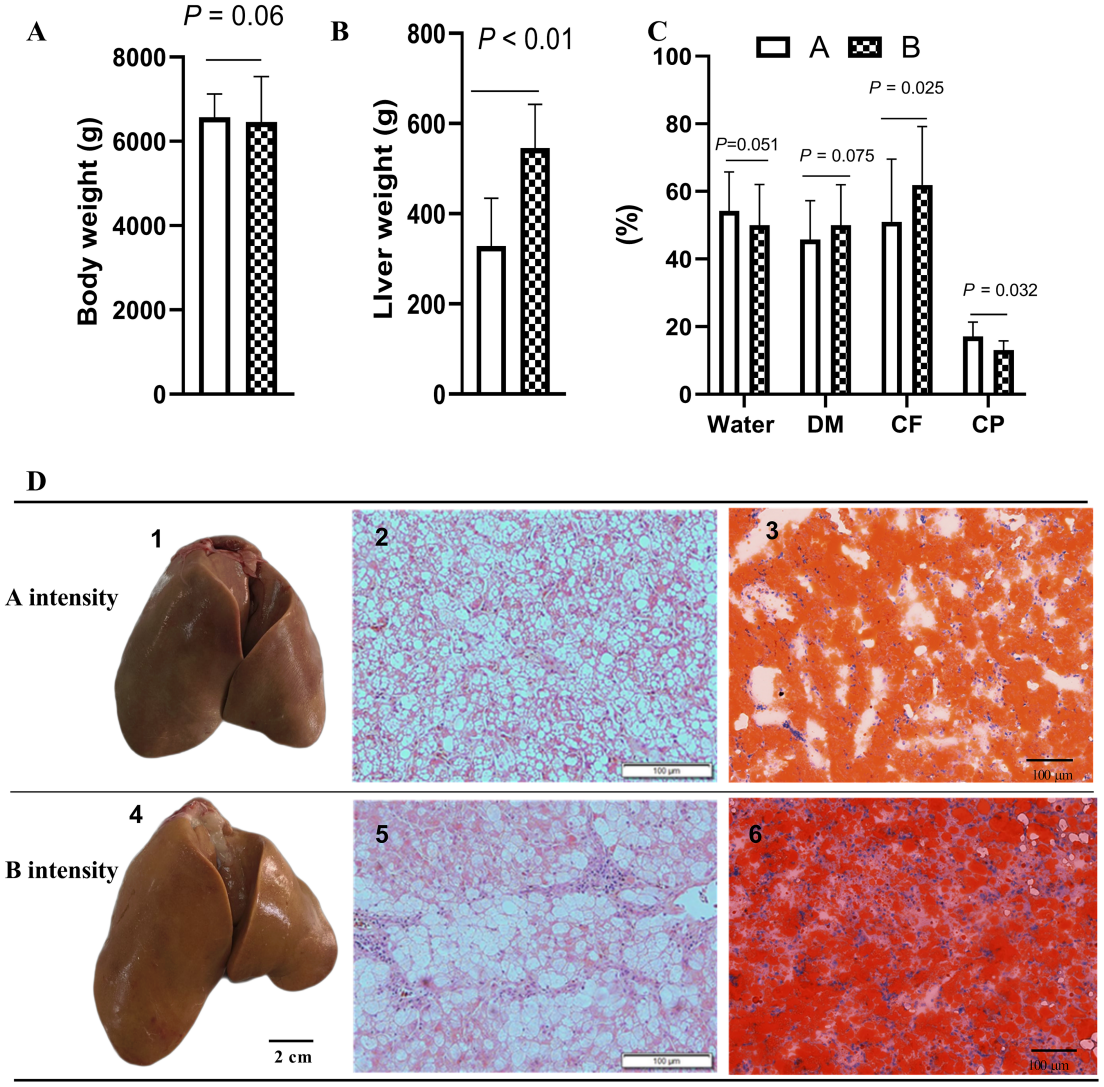
Influence of different force-feeding intensities on liver lipid deposition in force-fed geese. **(A)** Comparison of body weight after force-feeding (*n* = 40). **(B)** Comparison of liver weight (*n* = 40). **(C)** Comparison of water content, crude fat, and crude protein of *foie gras* (*n* = 40). **(D)** Comparison of *foie gras* and liver tissue sections of geese force-fed with different force-feeding intensity (*n* = 3); 1 and 4, geese fatty liver; 2 and 5, liver tissue sections (HE staining, 100x); 3 and 6, liver tissue sections (Oil Red O staining, 200x). Values are means ± SD. Different lowercase letters in the same set indicate differences among treatments at *p* < 0.05. CP, Coarse protein; CF, Crude fat; DM, Dry matter. **A** and **B** represent two different force-feeding intensities, respectively. An intensity was that geese were force-fed 4 times per day and lasting 28 days; B intensity was that geese were force-fed 5 times per day and lasting 18 days.

After force-feeding, the liver samples of geese force-fed with different force-feeding intensities were collected and weighed. No significant difference was observed in body weight between the A and B groups after force-feeding (*p* > 0.05) (Figure 1A). Compared with A intensity, B intensity significantly increased the weight of *foie gras* (*p* < 0.05) (Figure 1B). The crude fat content of the B group was higher than that of the A group (*p* < 0.05). The crude protein content of the A group was higher than that of the B group (*p* < 0.05). No significant difference was observed in water content between A and B, these two force-feeding intensity groups (*p* > 0.05) (Figure 1C). Figure 1D shows the influence of different force-feeding intensities on liver lipid deposition in force-fed geese from liver and liver tissue slices. The liver yellowness of the B group was higher than that of the A group. Liver tissue slices stained with HE showed that more fat accumulated in the liver of the B group. Especially, liver tissue slices stained with Oil Red O clearly showed that more lipid droplets were deposited in the hepatocytes of the B group. The results of the influence of different force-feeding intensities on serum parameters are shown in Table 1. The TG level of the B group was significantly higher than that of the A group (*p* < 0.05). The CHO level of the B group was significantly lower than that of the A group (*p* < 0.05). Compared with the A group, the B group had significantly higher levels of insulin and blood sugar (*p* < 0.05). The HDL level of the A group was significantly higher than that of the B group (*p* < 0.05), and the VLDL level of the B group was significantly higher than that of the A group (*p* < 0.05) (Table 2).

**Table 1:** Comparison of serum parameters of geese force-fed with different force-feeding intensities.

	A	B	*p*-value
Insulin (mIU/L)	57.95 ± 10.21	67.76 ± 8.43	<0.0001
Glucose (mmol/L)	11.00 ± 3.55	19.58 ± 5.4	<0.001
TG (mmol/L)	7.79 ± 3.51	11.09 ± 2.82	<0.0001
CHO (mmol/L)	31.5 ± 14.43	13.33 ± 9.62	<0.001
HDL (mmol/L)	11.62 ± 3.79	5.96 ± 2.77	<0.001
VLDL (mmol/L)	5.37 ± 2.45	8.03 ± 3.97	0.02

Values are means ± SD (*n* = 20). *p* < 0.05 represents a significant difference. TG, triglyceride; T-CHO, total cholesterol; HDL, high-density lipoprotein; VLDL, very low-density lipoprotein. A and B represent two different force-feeding intensities, respectively. An intensity was that geese were force-fed 4 times per day and lasting 28 days; B intensity was that geese were force-fed five times per day and lasting 18 days.

**Table 2:** Comparison of liver amino acids of geese force-fed with different force-feeding intensities (area ratio, %).

	A	B	*p*-value
Asp	3.84 ± 1.67	0.66 ± 0.3	<0.001
Glu	10.23 ± 3.11	3.32 ± 1.16	<0.001
Ser	7.25 ± 1.55	4.73 ± 1.57	<0.001
His	1.77 ± 0.4	0.65 ± 0.13	<0.001
Gly	8.99 ± 1.62	12.17 ± 3.62	0.004
Thr	4.62 ± 1.28	1.02 ± 0.26	<0.001
Arg	2.83 ± 0.74	1.74 ± 0.36	<0.001
Ala	14.04 ± 3.94	5.38 ± 2.21	<0.001
Tyr	2.25 ± 1.1	0.6 ± 0.37	<0.001
Lys	13.84 ± 2.17	54.89 ± 8.14	<0.001
Cys	/	/	/
Val	6.64 ± 1.58	1.41 ± 0.38	<0.001
Met	5.23 ± 0.89	6.44 ± 1.9	0.033
Phe	2.53 ± 0.69	0.57 ± 0.2	<0.001
Ile	2.99 ± 0.85	0.39 ± 0.34	<0.001
Leu	6.18 ± 1.54	2.56 ± 0.5	<0.001
Pro	3.27 ± 0.71	5.77 ± 1.58	<0.001

Values are means ± SD. A group (*n* = 15), B group (*n* = 20). *p* < 0.05 represents a significant difference. A and B represent two different force-feeding intensities, respectively. An intensity was that geese were force-fed four times per day and lasting 28 days; B intensity was that geese were force-fed five times per day and lasting 18 days. Cysteine is a sulfur-containing amino acid that is easily destroyed under acid hydrolysis conditions; its measured value is inaccurate and therefore omitted. “/” means null value in the Table.

### Liver transcriptome reveals the *foie gras* performance difference induced by different force-feeding intensities

**Figure 2:**
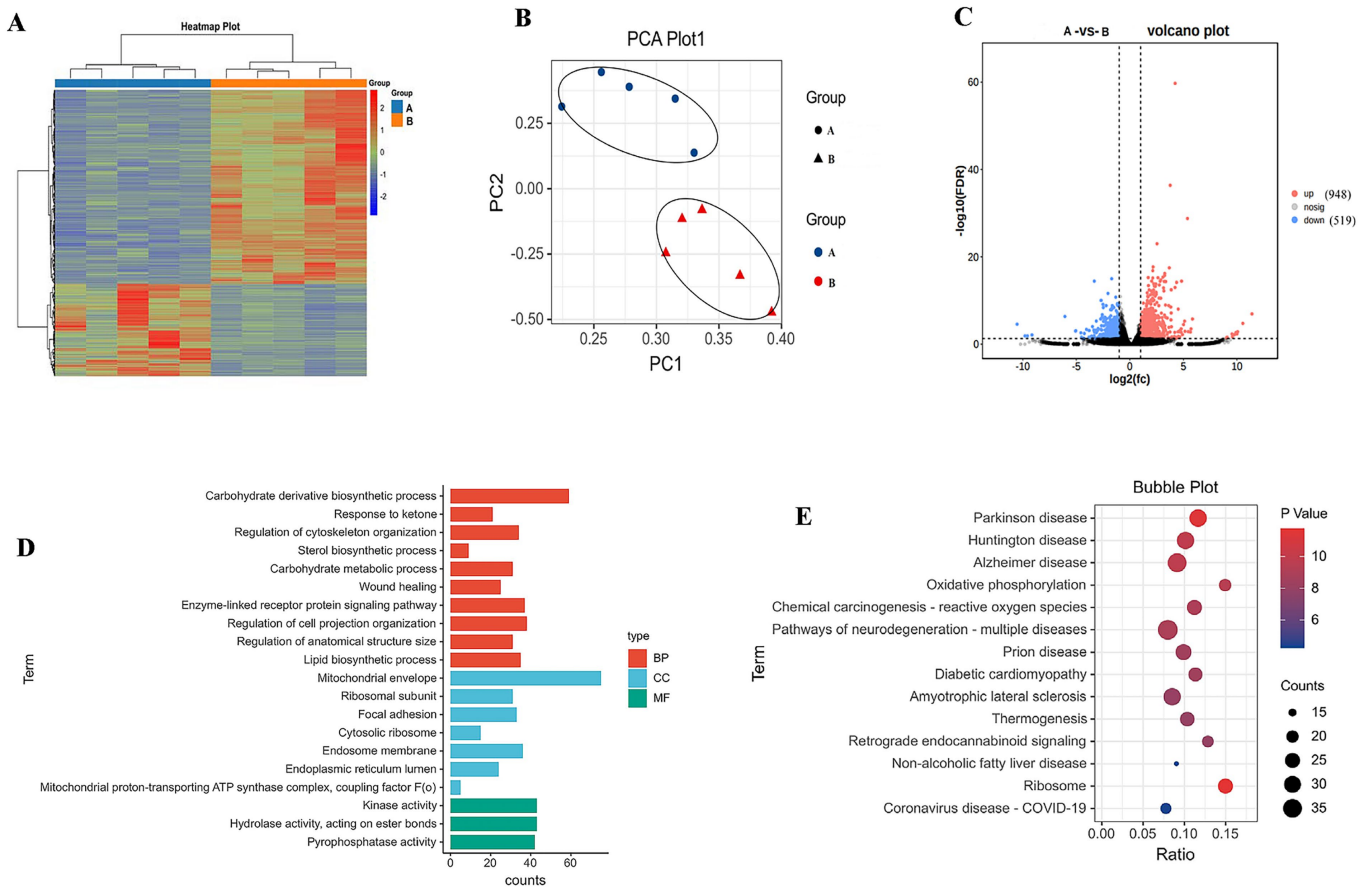
Study on the influence of different force-feeding intensities on *foie gras* performance from liver transcriptome (*n* = 5). **(A)**, Clustering heat map of differentially expressed genes (**A** *vs* **B**). **(B)**, Principal component analysis (PCA) of differentially expressed genes (**A** *vs* **B**). **(C)**, Volcano plots of differentially expressed genes (**A** *vs* **B**). **(D)**, KEGG analysis. **(E)**, GO analysis. **A** and **B** represent two different force-feeding intensities, respectively. An intensity was that geese were force-fed 4 times per day and lasting 28 days; **B** intensity was that geese were force-fed 5 times per day and lasting 18 days.

Figure 2 depicts the results on the influence of different force-feeding intensities on *foie gras* performance from the liver transcriptome. The liver samples of geese force-fed with different force-feeding intensities were collected and subjected to transcriptome sequencing. The gene expression level was represented by RPKM, and the RPKM value for each gene was calculated. According to the expression profiles analysis, the clustering diagram and PCA can directly demonstrate the distance and difference between samples. In the present study, hierarchical cluster analysis and PCA showed the distance and difference between liver samples of geese force-fed with different force-feeding intensities (Figures 2A,B). After screening with the standard (|log2FoldChange| > 1 and *p* < 0.05), the DEGs were identified between the A group and the B group (A *vs.* B). A total of 1,467 DEGs were obtained. Volcano plots showed that there were 948 upregulated DEGs and 519 downregulated DEGs (Figure 2C). DEGs involved in cell cycle, metabolism, and biosynthesis were presented in the Supplementary Table S1. The gene expression levels of key DEGs involved in glucolipid metabolism (*G6PD*, *IGF1*, *IGF2*, and MLX) were upregulated. The gene expression levels of key DEGs involved in the cell cycle (TP53) were upregulated, whereas the gene expression level of liver lipoprotein lipase (***LPL***) was downregulated. Further, the gene expression levels of key DEGs involved in insulin signal transduction (*IRS1*, *IRS4*, and *IGF1R*) were downregulated. In order to further investigate the biological functions and pathways enrichment of DEGs, GO, and KEGG analyses were performed. GO functional annotation showed that DEGs were enriched in 20 processes, which included three processes related to molecular function, seven processes related to cellular components, and 10 processes related to biological processes. The main enrichment GO processes include the biosynthesis process of carbohydrate derivatives, mitochondrial envelope, kinase activity, hydrolase activity (acting on ester bond), pyrophosphatase activity, regulation of nuclear body membrane, cytoskeleton tissue, regulation of enzyme-linked receptor protein signal pathway, regulation of cell projection tissue, and other biological processes (Figure 2D). KEGG analysis showed that DEGs were primarily enriched in these pathways: Parkinson’s disease, oxidative phosphorylation, retrograde endogenous cannabinoid signal, ribosome, diabetes cardiomyopathy, chemical carcinogenesis, reactive oxygen species, and other signal pathways (Figure 2E).

### Liver non-targeted amino acids metabolome reveals the *foie gras* performance difference induced by different force-feeding intensities

**Figure 3:**
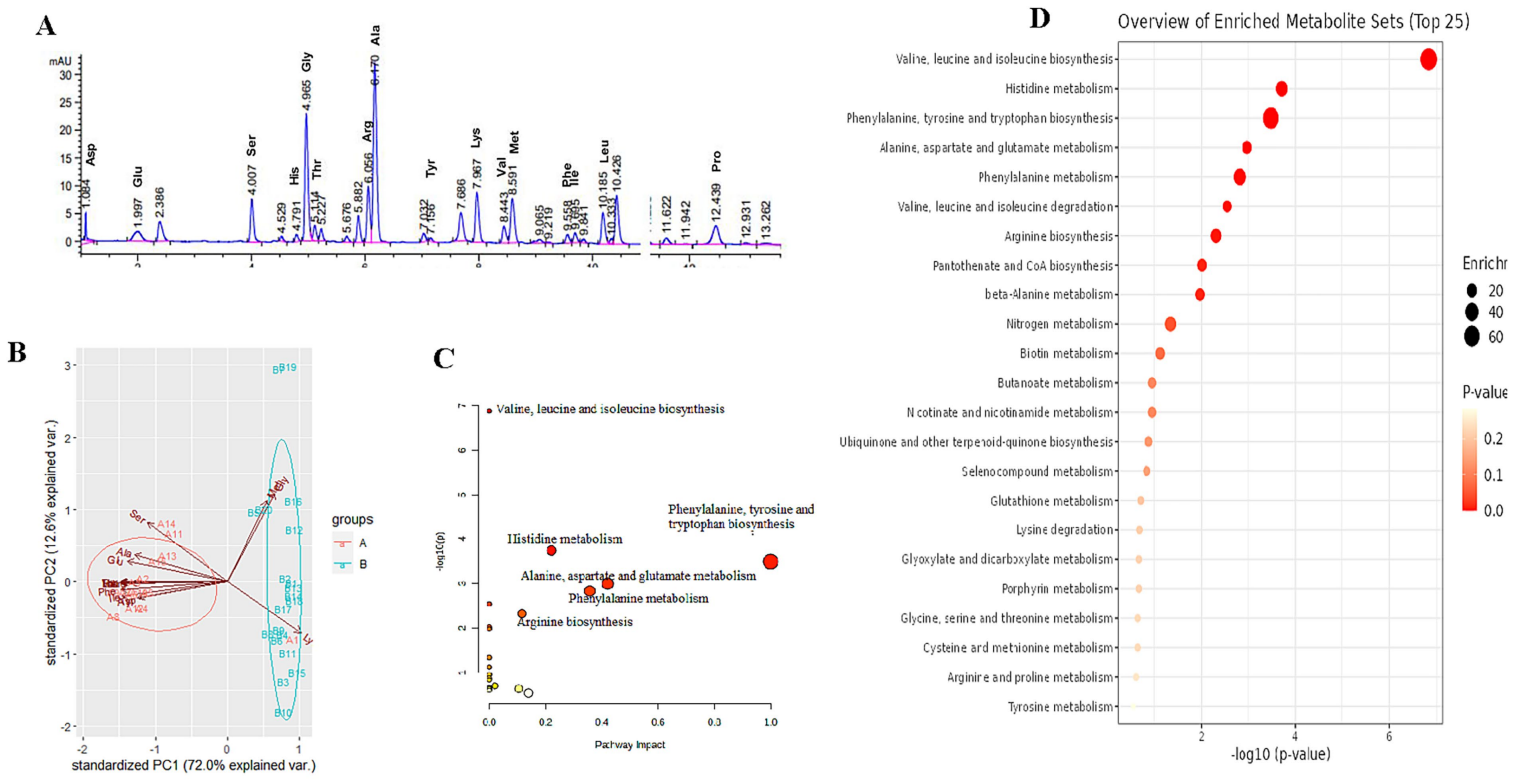
Study on the influence of different force-feeding intensities on *foie gras* performance from liver amino acid metabolome analysis (**A** *vs* **B**) [**(A)**, *n* = 15; **(B)** *n* = 20]. **(A)** Amino acid HPLC digital fingerprint. **(B)** Liver amino acids profile analysis - Principal component analysis (PCA). **(C)** Pathway impact of significant metabolic pathways. **(D)** Enrichment analysis map. **(C,D)** these two view maps were generated from MetaboAnalyst6.0. **A** and **B** represent two different force-feeding intensities, respectively. An intensity was that geese were force-fed four times per day and lasting 28 days; **B** intensity was that geese were force-fed five times per day and lasting 18 days.

Online derivatization was used for the determination of free amino acids in liver using reversed-phase HPLC. As shown in the free amino acid chromatogram, primary amino acids (16 amino acids) were derivatized with OPA (*λ* = 338 nm), and secondary amino acid (proline) was derivatized with FMOC (*λ* = 262 nm) (Figure 3A). As shown in Table 3, the comparison of liver free amino acids of geese force-fed with different force-feeding intensities was performed. Compared with the B group, the A group was significantly higher in the levels of Asp., Glu, Ser, His, Gly, Thr, Arg, Ala, Tyr, Val, Met, Phe, Ile, Leu, and Pro. The Lys level of the B group was significantly higher than that of the A group (*p* < 0.05). In order to better understand the classification and higher level of group separation, the PCA model was used to clarify the different amino acid patterns. The clear separation and discrimination were presented in PCA (Figure 3B), which suggested that different force-feeding intensities induced differences in the liver amino acid pattern. To further explore how the liver responds to different amino acids in response to different force-feeding intensities, MetaboAnalyst6.0-pathway analysis part (see text footnote 3, respectively) was used for pathway analysis and enrichment analysis. Based on the pathway impact scores and -ln *p*-value (Supplementary Table S2), the primary metabolic pathways were screened and visualized in the amino acid metabolome view map (Figure 3C). These pathways were mainly involved in the biosynthesis of valine, leucine, and isoleucine, histidine metabolism, phenylalanine, tyrosine, and tryptophan biosynthesis, alanine, aspartate, and glutamate metabolism, phenylalanine metabolism, valine, leucine, and isoleucine degradation, arginine biosynthesis, pantothenate and CoA biosynthesis. Alanine, aspartate, and glutamate metabolism and phenylalanine metabolism were the highest impact score pathways. Enrichment analysis showed that the top five significantly enriched KEGG pathways included valine, leucine, and isoleucine biosynthesis, histidine metabolism, phenylalanine, tyrosine, and tryptophan biosynthesis, and alanine, aspartate, and glutamate metabolism and phenylalanine metabolism (Figure 3D).

**Table 3:** Comparison of liver long-chain fatty acids of geese force-fed with different force-feeding intensities (g/100 g).

	A	B	*p*-value
SFA	9.01 ± 4.65	40.61 ± 9.19	<0.001
MUFA	5.72 ± 4.57	34.61 ± 8.27	<0.001
PUFA	4.33 ± 1.65	3.46 ± 1.08	0.068
UFA	10.06 ± 3.73	38.07 ± 8.91	<0.001
UFA/SFA	1.18 ± 0.17	0.94 ± 0.085	<0.001
C14:0	0.07 ± 0.11	1.02 ± 0.36	<0.001
C16:1	0.61 ± 0.68	6.44 ± 2.24	<0.001
C16:0	4.67 ± 2.78	24.01 ± 6.08	<0.001
C18:2n6c	1.15 ± 0.44	1.68 ± 0.86	0.037
C18:1n9c	5.1 ± 3.95	28.16 ± 6.58	<0.001
C18:0	4.26 ± 1.93	15.58 ± 3.25	<0.001
C20:4n6	2.99 ± 1.25	1.79 ± 0.73	0.001
C22:6n3	0.2 ± 0.18	0 ± 0	<0.001

Values are means ± SD. A group (*n* = 15), B group (*n* = 20). *p* < 0.05 represents a significant difference. A and B represent two different force-feeding intensities, respectively. An intensity was that geese were force-fed four times per day and lasting 28 days; B intensity was that geese were force-fed five times per day and lasting 18 days. MUFA, polyunsaturated fatty acid; PUFA, monounsaturated fatty acid; UFA, unsaturated fatty acids; SFA, saturated fatty acid.

### Targeted long-chain fatty acids metabolome reveals the *foie gras* performance difference induced by different force-feeding intensities

**Figure 4:**
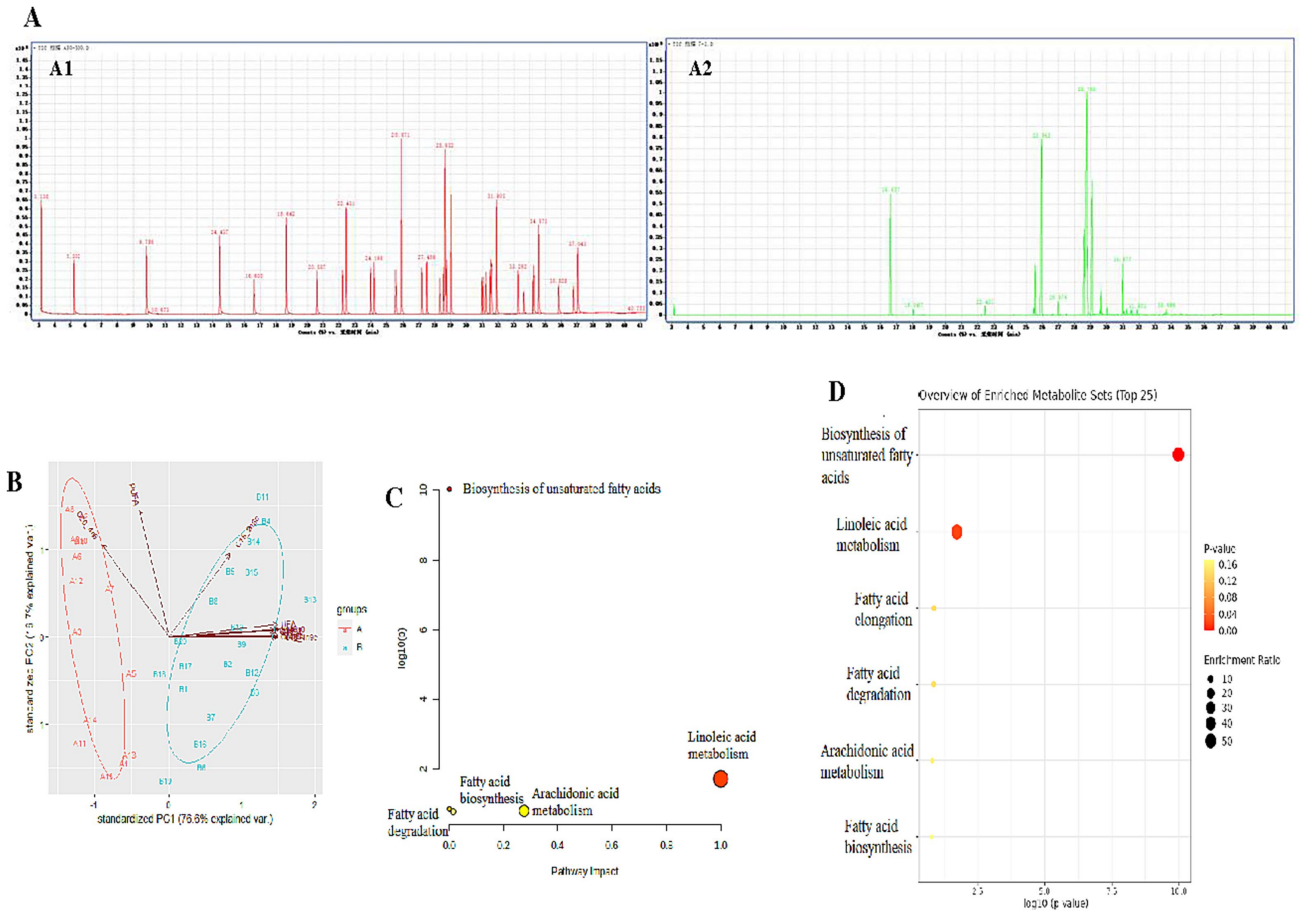
Study on the influence of different force-feeding intensities on *foie gras* performance from liver long-chain fatty acids profile analysis (**A** *vs* **B**) [**(A)** *n* = 15; **(B)** *n* = 20]. **(A)** Long-chain fatty acids digital fingerprint (A1, long-chain fatty acids standard sample; A2, liver sample). **(B)** Long-chain fatty acids profile analysis - principal component analysis (PCA). **(C)** Pathway impact of significant metabolic pathways. **(D)** Enrichment analysis of long-chain fatty acids. **(C,D)** these two view maps were generated from MetaboAnalyst6.0. **A** and **B** represent two different force-feeding intensities, respectively. An intensity was that geese were force-fed 4 times per day and lasting 28 days; **B** intensity was that geese were force-fed 5 times per day and lasting 18 days.

The long-chain fatty acids in *foie gras* were separated by GC–MS. The chromatogram scanning showed that long-chain fatty acids could be well separated (Figure 4A). The comparison results of liver long-chain fatty acids of geese force-fed with different force-feeding intensities are presented in Table 3. Compared with the A group, the B group had higher levels of SFA, MUFA, UFA, C14:0, C16:1, C16:0, C18:2n6c, C18:1n9c, and C18:0 (*p* < 0.05). The levels of C20:4n6 and C22:6n3 of the A group were higher than those of the B group (*p* < 0.05). No significant difference was observed between the A and B groups in the level of PUFA (*p* > 0.05). The ratio of UFA to SFA of the A group was higher than that of the B group (*p* < 0.05). To further reveal the differences in long-chain fatty acid profiles between the A and B groups, PCA was performed (Figure 4B). PCA presented the clear separation and discrimination between these two groups, which suggested that different force-feeding intensities induced differences in the liver fatty acids pattern. In addition, SFA, MUFA, UFA, C14:0, C16:1, C16:0, C18:2n6c, C18:1n9c, and C18:0 had a greater contribution to the B group, and C20:4n6 had a greater contribution to the A group. Both Pathway and enrichment analyses were performed to investigate further how the liver responds to different fatty acids in response to different force-feeding intensities (Supplementary Table S3). Visualization results from MetaboAnalyst6.0 software showed that pathways involved different fatty acids enriched biosynthesis of unsaturated fatty acids, linoleic acid metabolism, fatty acid elongation, fatty acid degradation, arachidonic acid metabolism, and fatty acid biosynthesis, and the highest impact score pathway was linoleic acid metabolism (Figures 4C,D).

### Study on the influence of different force-feeding intensities on *foie gras* performance from integrative analysis of transcriptome and metabolome

**Figure 5:**
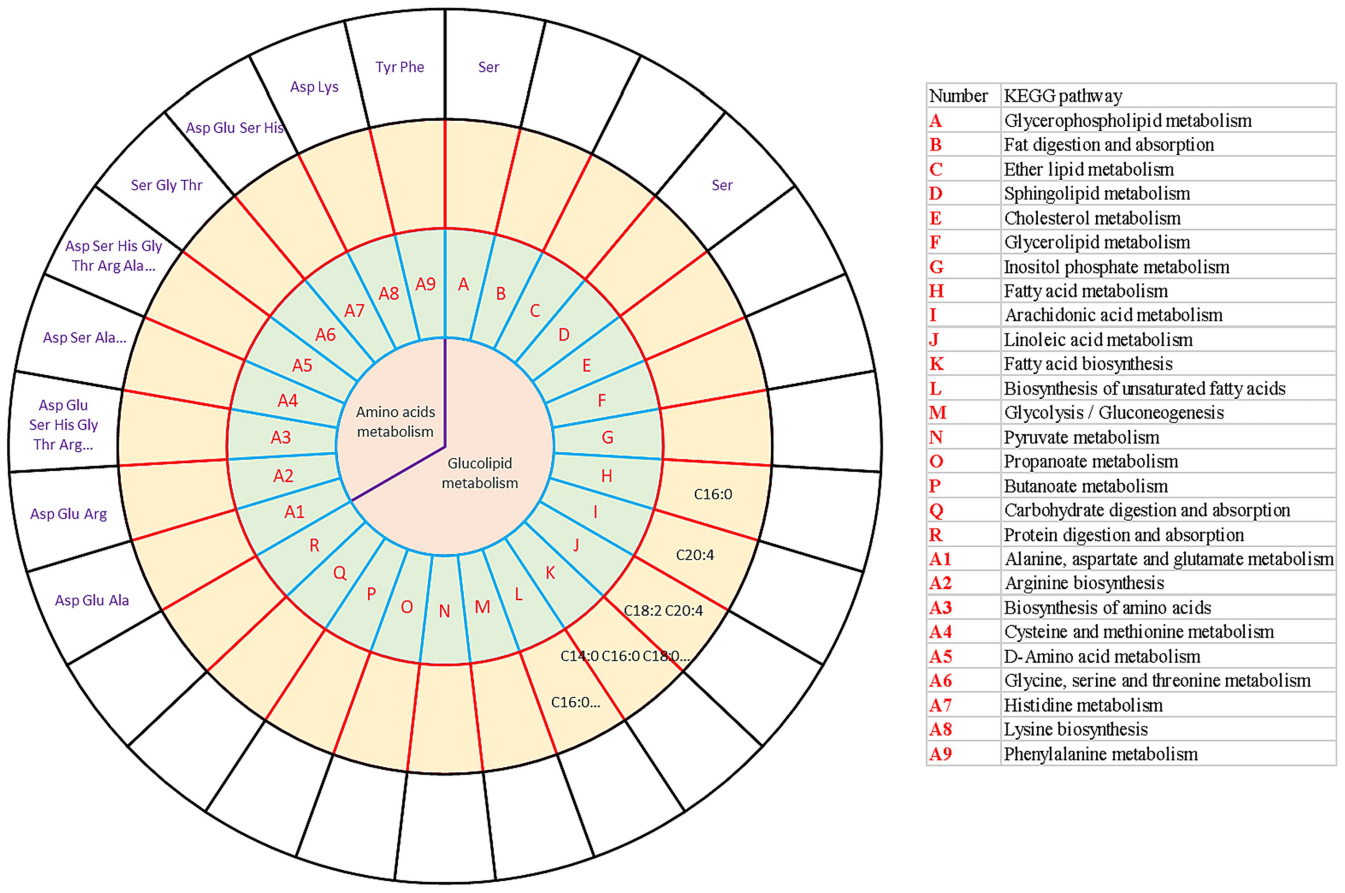
Integrative analysis of transcriptome and metabolome (amino acid metabolome and long-chain fatty acids metabolome) reveals the influence of different force-feeding intensities on *foie gras* performance. The circles from the center outward represented the first-tier pathways (the first circle); KEGG pathways (the second circle) involved in DEGs (**A** *vs* **B**); long-chain fatty acids metabolome shared the same pathways with the DEGs from liver transcriptome analysis (the third circle); amino acids metabolome shared the same pathways with the DEGs from liver transcriptome analysis (the fourth circle), respectively. The table on the right illustrates the KEGG pathways (the second circle) involved in DEGs (**A** *vs* **B**). **A** and **B** represent two different force-feeding intensities, respectively. An intensity was that geese were force-fed four times per day and lasting 28 days; B intensity was that geese were force-fed five times per day and lasting 18 days.

To more comprehensively present the interaction between the transcriptome, amino acids, and long-chain fatty acids, which responded to different force-feeding intensities, a conjoint analysis involving transcriptome and metabolome was performed. The KEGG database^4^ was used to search for the related KEGG pathways of the fatty acids and amino acids. KEGG pathways involved in metabolism, transcriptome, amino acids metabolome, and long-chain fatty acids metabolome were integrated (Figure 5). As shown in the results, amino acids mainly participated in amino acid metabolism, such as alanine, aspartic acid, glutamic acid metabolism, arginine biosynthesis, amino acid biosynthesis, cysteine and methionine metabolism, dextran amino acid metabolism, glycine, serine, threonine metabolism, histidine metabolism, lysine biosynthesis, and phenylalanine metabolism. In addition, not only did serine mediate amino acid metabolism, but also glycerol phospholipid metabolism and sphingolipid metabolism (lipid metabolism). Fatty acids mainly mediate lipid metabolism pathways. C16:0 mediated the biosynthesis of unsaturated fatty acids and fatty acid metabolism. C18:2 and C20:4 participated in linoleic acid metabolism. C20:4 mediated arachidonic acid metabolism. C14:0, C16:0, and C18:0 mediated the biosynthesis of fatty acids. Liver transcriptome, amino acids metabolome, and long-chain fatty acids metabolome collectively responded to different force-feeding intensities, and co-shaped *foie gras* performance difference induced by different force-feeding intensities.

## Discussion

Unlike mammals, waterfowl’s primary site of lipid synthesis is the liver, and the lipids of waterfowl are mainly endogenous and synthesized in the liver. The lipids produced in the liver of waterfowl are mostly transported by VLDL, and the lipids from the diet are mainly transported by chylomicrons. A majority of the chylomicrons and lipids transported by VLDL are deposited in peripheral adipose tissue and muscle after being hydrolyzed by LPL in the blood. Nevertheless, if the transported lipid by lipoprotein is not hydrolyzed by LPL, it will be re-transported to the liver and deposited there through specific lipoprotein receptors in the liver, thus facilitating lipid accumulation in the liver (13). In *foie gras* production, the geese are force-fed with a large number of high-energy diets, and a large quantity of glucose is used for TG synthesis in the liver. Thus, the synthesized fatty acid far exceeds the degraded fatty acid *via β*-oxidation, and the rate of TG production far exceeds the rate of TG ex-transportation, which results in a large amount of lipid accumulation in the liver and *foie gras* formation. In the present study, the serum parameters and DEGs responded to the differences in *foie gras* performance induced by different force-feeding intensities. The differences in *foie gras* performance and the levels of glucose, TG, and insulin between groups A and B align with the previous conclusion that force-fed geese had higher levels of glucose, TG, and insulin in serum; the force-fed geese showed better *foie gras* performance (20). The levels of CHO and HDL of the B group were significantly lower than those of the A group. The levels of HDL and CHO in plasma showed the same tendency in A and B, two force-feeding intensities, which was consistent with the fact that the CHO was transported using HDL as a carrier in plasma (22). The growth of peripheral adipose tissue and fattening of poultry are primarily dependent on the amount and utilization of TG carried by VLDL in plasma. Compared with A force-feeding intensity, B force-feeding intensity down-regulated the gene expression level of *LPL*. This result indicated that peripheral tissues may not easily use TG transported by lipoprotein and will be re-transported to the liver for deposition. A previous study showed that overfeeding down-regulated the gene expression level of *LPL*, thus facilitating the lipid accumulation in the liver of overfed geese (23). One of the reasons for *foie gras* formation may be that the rate of TG synthesis far exceeds the VLDL-TG assembly and secretion in goose liver. The comparison of susceptibility to liver steatosis in two breeds of geese showed that Landes Geese with stronger lipid production ability had lower plasma VLDL concentration and less lipid transport out of the liver after overfeeding; however, Polish Geese with stronger lipid production ability had higher plasma VLDL concentration and more lipid transport out of the liver after force-feeding (22). The results for ducks were the same as those for geese (24). However, in the present study, the serum VLDL level of the B group was higher, which seemingly contradicts the research above. The reason may be due to differences in liver lipid synthesis. The geese showed stronger liver lipid synthesis in B force-feeding intensity, and had stronger VLDL-TG assembly and secretion. A similar result was reported by Liu et al. (25), where Landaise geese with higher *foie gras* performance also had higher plasma VLDL concentration compared with Xupu Geese after overfeeding.

Amino acids are not only the basic units of proteins, but also important signal molecules that regulate glucolipid metabolism and energy balance. Thus, amino acid metabolism is of great significance to *foie gras* formation. Given the similarity between non-alcoholic fatty liver disease (NAFLD) and goose fatty liver formation (13), the theory of “gut-liver axis” (26) not only attracted a great deal of attention for disease research, which involves gut and NAFLD, but also was applied to explain the goose fatty liver formation. Metabolomics study reveals DON-induced intestinal toxicity affecting systemic metabolism (27). Wei et al. (28) reported that dietary amino acids have an impact on the microbiome. With the continuous fat accumulation in *foie gras* formation, the content of proteins and amino acids will continuously decrease per unit weight of liver. In the present study, the liver of the B group showed more severe hepatic steatosis; in contrast, the contents of live amino acids were lower than those of the A group, except for lysine. Intriguingly, the level of lysine was up-regulated in the B group, suggesting that lysine was a potential biomarker for severe hepatic steatosis. Serine-mediated fatty acid metabolism, whose metabolite is pyruvate, involves pyruvate in participating in the tricarboxylic acid (TCA) cycle (29), which aligns with the conjoint analysis of the current study. Conjoint analysis between transcriptome and metabolome (amino acids metabolome and fatty acids metabolome) indicated that Ser participated in glycerol phospholipid metabolism and sphingolipid metabolism. These changes collectively contributed to the development of hepatic steatosis and altered metabolic pathways in force-fed geese, which was consistent with findings in other livestock models where dietary interventions modulated intestinal development and metabolomic profiles (30). As said above, the process of *foie gras* formation was closely connected with hepatocyte enlargement and proliferation as well as endoplasmic reticulum stress (ERS) and insulin resistance (IR). Compared with A force-fed intensity, B force-fed intensity upregulated the gene expression of DEGs TP53. Our previous studies had demonstrated that TP53 regulated cell proliferation, and its overexpression promoted the proliferation of goose primary hepatocytes (31, 32). As IR, transcriptome analysis showed the gene expression levels of key DEGs involved in insulin signal transduction (*IRS1*, *IRS4*, and *IGF1R*) were downregulated, and the expression levels of *IGF1* and *IGF2* were upregulated, which suggested that B force-fed intensity induced more drastic IR (33). Branched-chain amino acids (BCAAs, leucine, isoleucine, and valine) metabolism is closely related to insulin resistance, and can lead to IR (34–36). Leucine is a ketogenic amino acid whose metabolites are acetoacetic acid and acetyl-CoA. Isoleucine is a glucogenic and ketogenic amino acid whose metabolite is propionyl-CoA. Valine is a glucogenic amino acid whose metabolite is succinyl-coenzyme A. Under normal circumstances, BCAAs enter the TCA cycle mainly through gluconeogenesis and ketogenesis, thus participating in the conversion between glucose, lipid, and protein. Qian et al. (37) contextualized transcriptional regulation tools. In obese patients and obese animal models, the activity of branched-chain ketoacid dehydrogenase (BCKD) (mainly in muscle and liver) decreased, which led to an elevation of circulating BCAAs levels (38, 39). Studies have shown that elevated circulating BCAAs regulate IR mainly by activating the mammalian target of rapamycin (mTOR) signaling pathway to regulate insulin signal transduction (40, 41). mTORC1 activity was regulated by BCAAs, especially leucine. After mTORC1 activation, serine phosphorylation of insulin receptor substrate 1 (IRS-1) was induced by activation of p70 ribosomal protein S6 kinase 1 (p70S6K1), which inhibited the activity of IRS-1, impaired insulin signal transduction, and induced IR (42). As described above, B force-fed intensity induced more drastic IR compared with A force-fed intensity. However, liver BCAAs levels of the B group were lower. Therefore, the relationship between circulating BCAAs and liver BCAAs needs further investigation. The individual and synergistic mechanisms of liver, muscle, and adipose tissue in coordinating systemic BCAAs metabolism leading to insulin resistance need to be further explored.

It is well known that *foie gras* formation is mainly because of the imbalance between fatty acid synthesis, transportation, and *β*-oxidation, leading to excessive fat accumulation in the liver of force-fed geese. Thus, fatty acid metabolism plays a crucial role in *foie gras* formation. The pentose phosphate pathway produces large amounts of NADPH, which provides a reducing agent for various synthetic reactions in cells, such as the synthesis of fatty acids and sterols. This process is regulated by glucose-6-phosphate dehydrogenase (G6PD) and 6-phosphogluconate dehydrogenase, which are two key enzymes (43). Compared with A force-feeding intensity, B force-feeding intensity up-regulated gene expression level of *G6PD*, which indicated that the pentose phosphate pathway will provide more raw materials for fatty acids synthesis in the liver of the B group. In addition, B force-feeding intensity induced a higher glucose level in plasma, which provided more raw materials for fatty acid synthesis in the liver. Correspondingly, *foie gras* fatty acids profile analysis showed that the contents of SFA and UFA of the B group were significantly higher than those of the A group. On the other hand, the increasing amount of unsaturated fatty acids in the liver of force-fed geese increased the tolerance to hepatic steatosis. It was reported that UFA could inhibit SFA-induced elevation of ceramides and inflammation (44). The fat composition of most mammals consists of saturated fatty acids. Studies have shown that increasing the content of saturated fatty acids changes the content of saturated phospholipids in cell membrane structure, destroys the structure of endoplasmic reticulum, and affects the fluidity of endoplasmic reticulum membrane, causing ERS (45). In contrast, *foie gras* is rich in unsaturated fatty acids, which can effectively reduce the lipid toxicity caused by the peroxidation of saturated fatty acids. The desaturation process of unsaturated fatty acids can effectively reduce ERS, mitochondrial damage, inflammation, and apoptosis of hepatocytes caused by IR, which is of great significance for maintaining the liver health of force-fed goose (46). In this current research, the levels of monounsaturated fatty acids (C16:1 and C18:1n9c) of the B group were higher than those of the A group. Stearoyl-CoA desaturase (SCD) is a key enzyme that catalyzes the formation of monounsaturated fatty acids (47). A previous study had demonstrated that *SCD* played a key role in goose fatty liver formation, which made the goose tolerant of a large amount of lipids deposited in the liver (23). In brief, B force-feeding intensity induced more unsaturated fatty acids synthesis in force-fed geese compared with A force-feeding intensity, and a larger amount of UFAs was associated with increased hepatic steatosis and lipid accumulation in the liver of force-fed geese.

## Conclusion

This is the first report describing the *foie gras* performance difference induced by different force-feeding intensities from multi-omics. Force-feeding five times per day and lasting 18 days contributed to higher levels of insulin, glucose, and TG in serum, which resulted in more fat accumulation in *foie gras* formation. Therefore, decreasing the force-feeding times per day and extending the force-feeding period cannot better promote liver lipid deposition in *foie gras* production. Conversely, increasing the force-feeding times per day not only achieved better *foie gras* performance but also shortened the force-feeding period. The DEGs involved in the insulin signaling pathway indicated more severe IR. Different unsaturated fatty acids mediated the biosynthesis of unsaturated fatty acids and fatty acid metabolism. They not only play a role in mediating amino acid metabolism through different free amino acids but also in lipid metabolism. In summary, the liver transcriptome, amino acids metabolome, and long-chain fatty acids metabolome collectively responded to different force-feeding intensities, and co-shaped the liver metabolism landscape difference, resulting from different force-feeding intensities.

## Data Availability

The original contributions presented in the study are publicly available. This data can be found here: https://doi.org/10.57760/sciencedb.27434.

## Footnotes

^1^ https://david.ncifcrf.gov/tools.jsp

^2^ http://bioinfo.org/kobas

^3^ https://www.metaboanalyst.ca/

^4^ https://www.genome.jp/kegg/

## References

[1] Hadinia SH Carneiro PRO Korver DR Zuidhof MJ. Energy partitioning by broiler breeder hens in conventional daily-restricted feeding and precision feeding systems. Poult Sci. (2019) 98:6721–32. doi: 10.3382/ps/pez387, PMID: 31265731 PMC8913959

[2] Wei R Ning R Han C Wei S Teng Y Li L. Lipidomics analysis reveals new insights into the goose fatty liver formation. Poult Sci. (2023) 102:102428. doi: 10.1016/j.psj.2022.102428, PMID: 36586388 PMC9811251

[3] Das R Mishra P Jha R. Feeding as a tool for improving performance and gut health of poultry: a review. Front Vet Sci (2021) 8:6. doi: 10.3389/fvets.2021.754246 PMC8632539 34859087

[4] Zhang T Li C Deng J Jia Y Qu L Ning Z. Chicken hypothalamic and ovarian DNA Methylome alteration in response to forced molting. Animals. (2023) 13:1012. doi: 10.3390/ani13061012, PMID: 36978553 PMC10044502

[5] Anene DO Akter Y Thomson PC Groves P O'Shea CJ. Effect of restricted feeding on hen performance, egg quality and organ characteristics of individual laying hens. Anim Nutr. (2023) 14:141–51. doi: 10.1016/j.aninu.2023.05.001, PMID: 37455791 PMC10338298

[6] Crouch AN Grimes JL Christensen VL Krueger KK. Effect of physical feed restriction during rearing on large white Turkey breeder hens: 2. Reproductive performance. Poult Sci. (2002) 81:16–22. doi: 10.1093/ps/81.1.16, PMID: 11885894

[7] Wei RX Ye FJ He F Song Q Xiong XP Yang WL. Comparison of overfeeding effects on gut physiology and microbiota in two goose breeds. Poult Sci. (2021) 100:100960. doi: 10.1016/j.psj.2020.12.057, PMID: 33652539 PMC7936201

[8] Wen Z Hou S Xie M Huang W Yu J. Amounts of force-feeding affect growth performance, serum biochemical parameters and liver histology of Pekin ducks. Chin J Anim Nutr. (2012) 24:69–77. doi: 10.3969/j.issn.1006-267x.2012.01.011

[9] Wen Z Zhu Y Tang J Xie M Huang W Yu J. Effects of force-feeding levels on carcass quality, body fat deposition and apparent digestibility of nutrients for Pekin ducks. Acta Vet Zootech Sin. (2012) 43:1247–54.

[10] Wen Z Xie M Fouad A Tang J Maqbool U Huang W. The effect of feed consumption levels on growth performance and apparent digestibility of nutrients in white Pekin ducks. J Appl Anim Res. (2015) 43:112–7. doi: 10.1080/09712119.2014.928624

[11] Wen Z Xie M Huang W Yu J Hou S. Effects of different force-feeding amounts on growth performance, carcass quality and body fat deposition for mule ducks. Acta Vet Zootech Sin. (2013) 44:419–26.

[12] Wen ZG Jiang Y Tang J Xie M Yang PL Hou SS. Effect of feed consumption levels on growth performance and carcass composition during the force-feeding period in foie gras production of male mule ducks. Animal. (2016) 10:1417–22. doi: 10.1017/s175173111600032x, PMID: 26948181

[13] Wei R Han C Deng D Ye F Gan X Liu H. Research progress into the physiological changes in metabolic pathways in waterfowl with hepatic steatosis. Br Poult Sci. (2020) 62:118–24. doi: 10.1080/00071668.2020.1812527, PMID: 32902307

[14] Zhang C Hou S Wang Y Liu F Xie M. Feed input and excreta collection time in metabolisable energy assays for ducks. Czeh J Anim Sci. (2007) 52:463–8. doi: 10.17221/2331-cjas

[15] Wei R Han C Wei S Teng Y Li L Liu H. Integrative analysis of transcriptome and lipidome reveals fructose pro-steatosis mechanism in goose fatty liver. Front Nutr. (2023) 9:9. doi: 10.3389/fnut.2022.1052600, PMID: 36704791 PMC9871465

[16] Luo Z Wei R Teng Y Ning R Bai L Lu C. Influence of different types of sugar on overfeeding performance-part of meat quality. Poult Sci. (2022) 101:102149. doi: 10.1016/j.psj.2022.102149, PMID: 36209604 PMC9547294

[17] Kim D Langmead B Salzberg SL. HISAT: a fast spliced aligner with low memory requirements. Nat Methods. (2015) 12:357–60. doi: 10.1038/nmeth.3317, PMID: 25751142 PMC4655817

[18] Sherman BT Hao M Qiu J Jiao X Baseler MW Lane HC. DAVID: a web server for functional enrichment analysis and functional annotation of gene lists (2021 update). Nucleic Acids Res. (2022) 50:W216–w221. doi: 10.1093/nar/gkac194, PMID: 35325185 PMC9252805

[19] Bu D Luo H Huo P Wang Z Zhang S He Z. KOBAS-i: intelligent prioritization and exploratory visualization of biological functions for gene enrichment analysis. Nucleic Acids Res. (2021) 49:W317–w325. doi: 10.1093/nar/gkab447, PMID: 34086934 PMC8265193

[20] Wei R Deng D Teng Y Lu C Luo Z Abdulai M. Study on the effect of different types of sugar on lipid deposition in goose fatty liver. Poult Sci. (2022) 101:101729. doi: 10.1016/j.psj.2022.101729, PMID: 35172237 PMC8850742

[21] Tang Y Horikoshi M Li WX. Ggfortify: unified interface to visualize statistical results of popular R packages. R J. (2016) 8:474–85. doi: 10.32614/rj-2016-060

[22] Fournier E Peresson R Guy G Hermier D. Relationships between storage and secretion of hepatic lipids in two breeds of geese with different susceptibility to liver steatosis. Poult Sci. (1997) 76:599–607. doi: 10.1093/ps/76.4.599, PMID: 9106888

[23] Lu LZ Chen Y Wang Z Li XF Chen WH Tao ZR. The goose genome sequence leads to insights into the evolution of waterfowl and susceptibility to fatty liver. Genome Biol. (2015) 16:89. doi: 10.1186/s13059-015-0652-y, PMID: 25943208 PMC4419397

[24] Hermier D Guy G Guillaumin S Davail S Andre J-M Hoo-Paris R. Differential channelling of liver lipids in relation to susceptibility to hepatic steatosis in two species of ducks. Comp Biochem Physiol B: Biochem Mol Biol. (2003) 135:663–75. doi: 10.1016/s1096-4959(03)00146-5, PMID: 12892758

[25] Liu X-y He R-g Huang C-s Li X Zhou Q-a Wang C. Hepatic lipogenesis associated with biochemical changes in overfed Landaise geese and China Xupu geese. Agric Sci China. (2006) 5:390–6. doi: 10.1016/S1671-2927(06)60066-7

[26] Miura K Ohnishi H. Role of gut microbiota and toll-like receptors in nonalcoholic fatty liver disease. World J Gastroenterol. (2014) 20:7381–91. doi: 10.3748/wjg.v20.i23.7381, PMID: 24966608 PMC4064083

[27] Wang Y Wang L Du Y Yao F Zhao M Cai C. Metabolomics study reveals DON-induced intestinal toxicity in adult zebrafish through disruption of amino acid metabolism and sphingolipid signaling pathway. Aquat Toxicol. (2025) 282:107324. doi: 10.1016/j.aquatox.2025.107324, PMID: 40112585

[28] Wei X Wu H Wang Z Zhu J Wang W Wang J. Rumen-protected lysine supplementation improved amino acid balance, nitrogen utilization and altered hindgut microbiota of dairy cows. Anim Nutr. (2023) 15:320–31. doi: 10.1016/j.aninu.2023.08.001, PMID: 38053803 PMC10694044

[29] Yu Y Lyu W Fu Z Fan Q Xiao Y Ren Y. Metabolic profiling analysis of liver in Landes geese during the formation of fatty liver via GC-TOF/MS. Front Physiol. (2022) 12:12. doi: 10.3389/fphys.2021.783498, PMID: 35046836 PMC8761942

[30] Bai M Liu H Yan Y Duan S Szeto IM-Y He J. Hydrolyzed protein formula improves the nutritional tolerance by increasing intestinal development and altering cecal microbiota in low-birth-weight piglets. Front Nutr. (2024) 11:11. doi: 10.3389/fnut.2024.1439110, PMID: 39555191 PMC11565607

[31] Han C Wei S Song Q He F Xiong X Wan H. Insulin stimulates goose liver cell growth by activating PI3K-AKT-mTOR signal pathway. Cell Physiol Biochem. (2016) 38:558–70. doi: 10.1159/000438650, PMID: 26845041

[32] Wei S Han C He F Song Q Kang B Liu H. Inhibition of PI3K-Akt-mTOR signal pathway dismissed the stimulation of glucose on goose liver cell growth. J Anim Physiol Anim Nutr. (2017) 101:e133–43. doi: 10.1111/jpn.12574, PMID: 27859698

[33] Wei R Han C. Insights into the influence of three types of sugar on goose fatty liver formation from endoplasmic reticulum stress (ERS). Poult Sci. (2024) 103:103466. doi: 10.1016/j.psj.2024.103466, PMID: 38277893 PMC10840336

[34] Vieira EES Pereira IC Braz AF Nascimento-Ferreira MV de Oliveira Torres LR Freitas Brito A. Food consumption of branched chain amino acids and insulin resistance: a systematic review of observational studies in humans. Clin Nutr ESPEN. (2020) 40:277–81. doi: 10.1016/j.clnesp.2020.09.007, PMID: 33183550

[35] Yu D Richardson NE Green CL Spicer AB Murphy ME Flores V. The adverse metabolic effects of branched-chain amino acids are mediated by isoleucine and valine. Cell Metab. (2021) 33:905–922.e6. doi: 10.1016/j.cmet.2021.03.025, PMID: 33887198 PMC8102360

[36] Zhou M Shao J Wu C-Y Shu L Dong W Liu Y. Targeting BCAA catabolism to treat obesity-associated insulin resistance. Diabetes. (2019) 68:1730–46. doi: 10.2337/db18-0927, PMID: 31167878 PMC6702639

[37] Qian F-C Zhou L-W Li Y-Y Yu Z-M Li L-D Wang Y-Z. SEanalysis 2.0: a comprehensive super-enhancer regulatory network analysis tool for human and mouse. Nucleic Acids Res. (2023) 51:W520–7. doi: 10.1093/nar/gkad408, PMID: 37194711 PMC10320134

[38] Hutson SM Sweatt AJ Lanoue KF. Branched-chain corrected amino acid metabolism: implications for establishing safe intakes. J Nutr. (2005) 135:1557S–64S. doi: 10.1093/jn/135.6.1557S, PMID: 15930469

[39] White PJ Lapworth AL An J Wang L McGarrah RW Stevens RD. Branched-chain amino acid restriction in Zucker-fatty rats improves muscle insulin sensitivity by enhancing efficiency of fatty acid oxidation and acyl-glycine export. Mol Metab. (2016) 5:538–51. doi: 10.1016/j.molmet.2016.04.006, PMID: 27408778 PMC4921791

[40] Lynch CJ. Role of leucine in the regulation of mTOR by amino acids: revelations from structure-activity studies. J Nutr. (2001) 131:861S–5S. doi: 10.1093/jn/131.3.861S, PMID: 11238775

[41] Nie C He T Zhang W Zhang G Ma X. Branched chain amino acids: beyond nutrition metabolism. Int J Mol Sci. (2018) 19:954. doi: 10.3390/ijms19040954, PMID: 29570613 PMC5979320

[42] Yoon M-S. The role of mammalian target of rapamycin (mTOR) in insulin signaling. Nutrients. (2017) 9:1176. doi: 10.3390/nu9111176, PMID: 29077002 PMC5707648

[43] Patra KC Hay N. The pentose phosphate pathway and cancer. Trends Biochem Sci. (2014) 39:347–54. doi: 10.1016/j.tibs.2014.06.005, PMID: 25037503 PMC4329227

[44] Legrand-Poels S Esser N L'Homme L Scheen A Paquot N Piette J. Free fatty acids as modulators of the NLRP3 inflammasome in obesity/type 2 diabetes. Biochem Pharmacol. (2014) 92:131–41. doi: 10.1016/j.bcp.2014.08.013, PMID: 25175736

[45] Ariyama H Kono N Matsuda S Inoue T Arai H. Decrease in membrane phospholipid unsaturation induces unfolded protein response. J Biol Chem. (2010) 285:22027–35. doi: 10.1074/jbc.M110.126870, PMID: 20489212 PMC2903364

[46] Geng T Xia L Li F Xia J Zhang Y Wang Q. The role of endoplasmic reticulum stress and insulin resistance in the occurrence of goose fatty liver. Biochem Biophys Res Commun. (2015) 465:83–7. doi: 10.1016/j.bbrc.2015.07.134, PMID: 26235878

[47] Bai YH McCoy JG Levin EJ Sobrado P Rajashankar KR Fox BG. X-ray structure of a mammalian stearoyl-CoA desaturase. Nature. (2015) 524:252–6. doi: 10.1038/nature14549, PMID: 26098370 PMC4689147

