# Supplementary material for: The integrative analysis of transcriptome and metabolome reveals differences in raw foie gras performance induced by different force-feeding intensities in male Tianfu meat geese
Source: Front Vet Sci. 2025 Sep 25;12:1653733. doi: 10.3389/fvets.2025.1653733 (PMC12507551; doi:10.3389/fvets.2025.1653733)
Supplement: Supplementary file 1 [file Table_1.DOCX]

**Supplementary materials**

**Table S1** Liver key differentially expressed genes (DEGs) (A *vs* B)

|  | Gene | Gene name | log2(fc) | PValue | FDR | pathway |
| --- | --- | --- | --- | --- | --- | --- |
| Up | G6PD | 6-phosphate glucose dehydrogenase | 2.38 | 9.6E-17 | 5E-14 | Pentose phosphate pathway |
|  | IGF1 | Insulin-like growth factor 1 | 3.12 | 0.00073 | 0.008 | insulin/insulin-like growth factor signaling |
|  | IGF2 | Insulin-like growth factor 2 | 1.53 | 2.9E-05 | 0.0006 | insulin/insulin-like growth factor signaling |
|  | MLX | MAX-like protein X | 1.31 | 6.7E-07 | 3E-05 | Insulin resistance, Non-alcoholic fatty liver disease |
|  | TP53 | tumor protein p53 | 1.05 | 0.00453 | 0.0329 | Cell cycle |
| Down | LPL | lipoprotein lipase | -1.13 | 0.00117 | 0.0115 | Glycolipid metabolism |
|  | IRS1 | insulin receptor substrate 1 | -1.41 | 0.00123 | 0.012 | Insulin resistance, Insulin signaling pathway |
|  | IRS4 | insulin receptor substrate 4 | -1.48 | 5E-07 | 2E-05 | Insulin resistance, Insulin signaling pathway |
|  | IGF1R | insulin-like growth factor 1 receptor | -1.42 | 0.00022 | 0.0031 | PI3K-Akt signaling pathway, mTOR signaling pathway, AMPK signaling pathway |

**Table S2** Liver amino acids metabolism pathway analysis.

|  | Total | Expected | Hits | Raw p | -LOG_10_(p) | Holm adjust | FDR | Impact |
| --- | --- | --- | --- | --- | --- | --- | --- | --- |
| Valine, leucine and isoleucine biosynthesis | 8 | 0.060952 | 4 | 1.33E-07 | 6.8746 | 1.07E-05 | 1.07E-05 | 0 |
| Histidine metabolism | 16 | 0.1219 | 3 | 0.000179 | 3.7466 | 0.01416 | 0.007169 | 0.22131 |
| Phenylalanine, tyrosine and tryptophan biosynthesis | 4 | 0.030476 | 2 | 0.000317 | 3.4992 | 0.024708 | 0.008447 | 1 |
| Alanine, aspartate and glutamate metabolism | 28 | 0.21333 | 3 | 0.000996 | 3.0019 | 0.076657 | 0.019911 | 0.42068 |
| Phenylalanine metabolism | 8 | 0.060952 | 2 | 0.001453 | 2.8376 | 0.11046 | 0.023254 | 0.35714 |
| Valine, leucine and isoleucine degradation | 40 | 0.30476 | 3 | 0.00285 | 2.5451 | 0.21378 | 0.038005 | 0 |
| Arginine biosynthesis | 14 | 0.10667 | 2 | 0.004605 | 2.3368 | 0.34075 | 0.052625 | 0.11675 |
| Pantothenate and CoA biosynthesis | 20 | 0.15238 | 2 | 0.009373 | 2.0281 | 0.6842 | 0.091692 | 0 |
| beta-Alanine metabolism | 21 | 0.16 | 2 | 0.010315 | 1.9865 | 0.7427 | 0.091692 | 0 |
| Nitrogen metabolism | 6 | 0.045714 | 1 | 0.044922 | 1.3475 | 1 | 0.35938 | 0 |
| Biotin metabolism | 10 | 0.07619 | 1 | 0.073835 | 1.1317 | 1 | 0.53698 | 0 |
| Butanoate metabolism | 15 | 0.11429 | 1 | 0.10885 | 0.96319 | 1 | 0.66982 | 0 |
| Nicotinate and nicotinamide metabolism | 15 | 0.11429 | 1 | 0.10885 | 0.96319 | 1 | 0.66982 | 0 |
| Ubiquinone and other terpenoid-quinone biosynthesis | 18 | 0.13714 | 1 | 0.12927 | 0.88851 | 1 | 0.73867 | 0 |
| Selenocompound metabolism | 20 | 0.15238 | 1 | 0.14264 | 0.84576 | 1 | 0.76075 | 0 |
| Glutathione metabolism | 28 | 0.21333 | 1 | 0.19428 | 0.71158 | 1 | 0.85746 | 0.01966 |
| Lysine degradation | 30 | 0.22857 | 1 | 0.20673 | 0.68459 | 1 | 0.85746 | 0 |
| Porphyrin metabolism | 31 | 0.23619 | 1 | 0.21289 | 0.67184 | 1 | 0.85746 | 0 |
| Glyoxylate and dicarboxylate metabolism | 32 | 0.24381 | 1 | 0.21901 | 0.65953 | 1 | 0.85746 | 0 |
| Glycine, serine and threonine metabolism | 33 | 0.25143 | 1 | 0.22508 | 0.64765 | 1 | 0.85746 | 0 |
| Cysteine and methionine metabolism | 33 | 0.25143 | 1 | 0.22508 | 0.64765 | 1 | 0.85746 | 0.10446 |
| Arginine and proline metabolism | 36 | 0.27429 | 1 | 0.24305 | 0.61431 | 1 | 0.88381 | 0 |
| Tyrosine metabolism | 42 | 0.32 | 1 | 0.27783 | 0.55622 | 1 | 0.96637 | 0.13972 |

**Table S3** Liver long-chain fatty acids metabolism pathway analysis.

|  | Total | Expected | Hits | Raw p | -LOG_10_(p) | Holm adjust | FDR | Impact |
| --- | --- | --- | --- | --- | --- | --- | --- | --- |
| Biosynthesis of unsaturated fatty acids | 36 | 0.13714 | 6 | 9.28E-11 | 10.033 | 7.42E-09 | 7.42E-09 | 0 |
| Linoleic acid metabolism | 5 | 0.019048 | 1 | 0.018927 | 1.7229 | 1 | 0.75708 | 1 |
| Fatty acid elongation | 39 | 0.14857 | 1 | 0.13988 | 0.85424 | 1 | 1 | 0 |
| Fatty acid degradation | 39 | 0.14857 | 1 | 0.13988 | 0.85424 | 1 | 1 | 0 |
| Arachidonic acid metabolism | 44 | 0.16762 | 1 | 0.15657 | 0.80529 | 1 | 1 | 0.27659 |
| Fatty acid biosynthesis | 47 | 0.17905 | 1 | 0.16645 | 0.7787 | 1 | 1 | 0.01473 |

**Long-chain fatty acids detection**


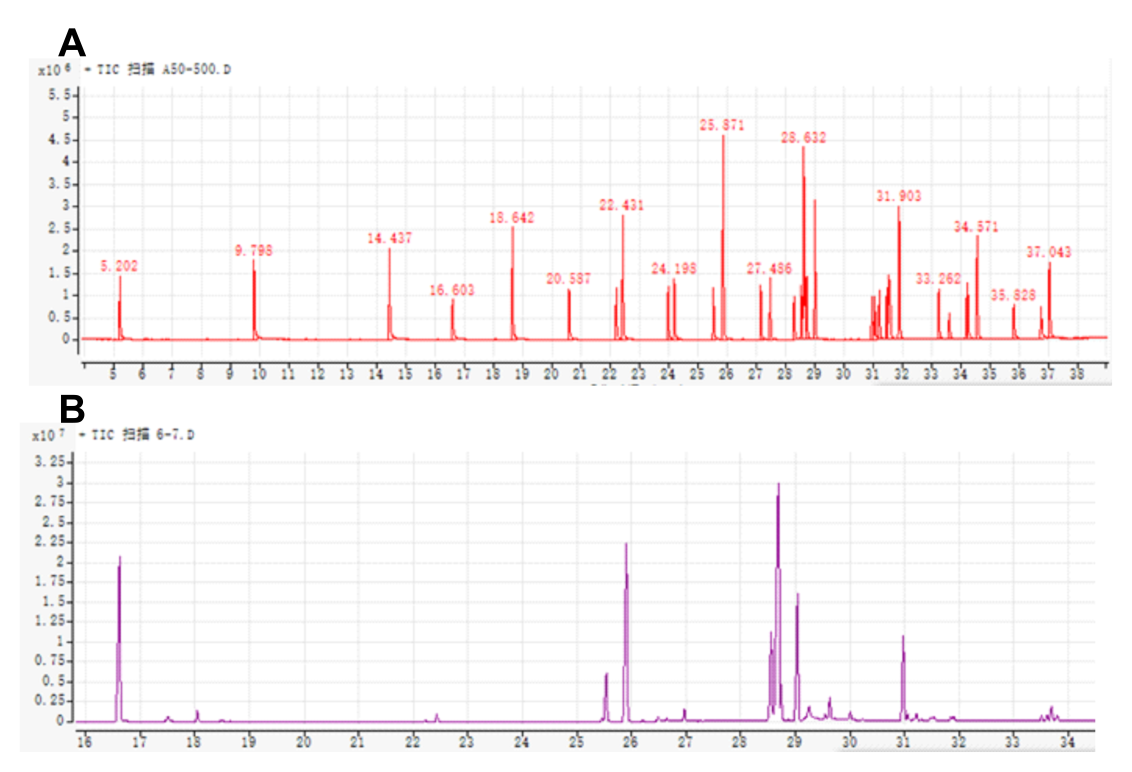


**Figure S1** Determination of fatty acids by gas chromatography. A, peak diagram of 37 fatty acid methyl esters; B, the peak of *foie gras* fatty acids. The corresponding relationship between retention time (RT) and the order of fatty acids peaks was showed in Table S4.

**Table S4** The corresponding relationship between retention time (RT) and the order of fatty acids peaks

| molecular formula | CAS | RT |
| --- | --- | --- |
| C15H30O2 | 124-10-7 | 22.431 |
| C17H32O2 | 1120-25-8 | 25.549 |
| C17H34O2 | 112-39-0 | 25.871 |
| C17H34O2 | 112-63-0 | 28.539 |
| C19H36O2 | 112-62-9 | 28.632 |
| C19H38O2 | 112-61-8 | 29.015 |
| C21H34O2 | 2566-89-4 | 30.969 |
| C23H34O2 | 2566-90-7 | 33.619 |

**Calculation of fatty acid concentration:**

The content of single fatty acid methyl ester in the sample was calculated according to formula (1):

X_i_ =F_i_ × $\frac{A_{i}}{A_{C11}}$ × $\frac{\rho_{c11}\times V_{C11}\times1.0067}{m}$ × 100 ……………………… (1);

Note: X_i_ - content of fatty acid methyl ester i in sample, unit = g/100g;

F_i_ - response factor of fatty acid methyl ester i;

A_i_ - peak area of fatty acid methyl ester i in samples;

A_C11_ - peak area of inner standard C11:0 which was added to the sample;

ρ_C11_ - content of inner standard C11:0, unit =mg/mL;

V _C11_ - volume of inner standard C11:0 which was added to the sample, unit =mL;

1.0067 - conversion coefficient of C11:0 to methyl undecarbonate;

m - weight of sample, unit = mg;

100 - conversion coefficient for converting the content to the content per 100g sample.

Response factor of fatty acid methyl ester i - F_i_ was calculated according to formula (2):

F_i_ = $\frac{\rho_{\mathrm{Si}}\times A_{11}}{A_{\mathrm{Si}}\times\rho_{11}}$………………………… (2);

Note: F_i_ - response factor of fatty acid methyl ester i;

ρ_Si_ - content of fatty acid methyl ester i in fatty acid methyl ester qualitative mixture, unit = mg/mL;

A_11_ - area of methyl undecarbonate;

A_Si_ - area of fatty acid methyl ester i;

ρ_11_ - content of methyl undecarbonate in mixed standard sample , unit = mg/mL;

C11:0 = Undecanoicacid,1,1',1''-(1,2,3-propanetriyl) ester (CAS#: 13552-80-2).

Saturated fat (acids) content of sample was calculated according to formula (3):

Single saturated fat (acids) content of sample was calculated according to formula (4):

X_Saturated Fat_ = ∑ X_SFAi_ ………………………… (3);

X_SFAi_ = X_FAMEi_ ×F_FAMEi - FAi_ ………………………… (4);

Note: X_Saturated Fat_ - saturated fat (acid) content, unit = g/100g;

X_SFAi_ - single saturated fat acid content, unit = g/100g;

X_FAMEi_ - single saturated fat acid methyl ester, unit = g/100g;

F_FAMEi-FAi_ - coefficient for fatty acid methyl esters were converted to fatty acids;

coefficient for fatty acid methyl esters were converted to fatty acids - F_FAMEi - FAi_ (S-Table 2).

Coefficient for fatty acid methyl esters were converted to fatty acids - F_FAMEi - FAi_ was calculated according to formula (5):

F_FAMEi - FAi_ = M_FAi_ / F_AMEi_ ………………………… (5)

Note: F_FAMEi - FAi_ - coefficient for fatty acid methyl esters convert to fatty acids;

M_FAi_ - molecular mass of fatty acids i;

F_AMEi_ ——— Molecular mass of fatty acid methyl esters i.

Mono- unsaturated fat acid content of sample was calculated according to formula (6):

single mono-unsaturated fat acid methyl esters content of sample was calculated according to formula (7):

X_Mono-UnsaturatedFat_ = ∑_XMUFAi_ ………………………… (6)

X_MUFAi_ = X_FAMEi_ × F_FAMEi-FAi_ ………………………… (7)

Note: X_Mono-UnsaturatedFat_ - mono-unsaturated fat acid content of sample, unit = g/100g;

X_MUFAi_ - single mono-unsaturated fat acid content in sample, unit = g/100g;

X_FAMEi_ - single mono-unsaturated fat acid methyl esters content in sample, unit = g/100g;

F_FAMEi-FAi_ - coefficient for fatty acid methyl esters were converted to fatty acids;

coefficient for fatty acid methyl esters were converted to fatty acids - F_FAMEi-FAi_ (S-Table 2).

Poly- unsaturated fat acid content of sample was calculated according to formula (8);

single poly-unsaturated fat acid methyl esters content of sample was calculated according to formula (9):

X_Poly-UnsaturatedFat_ = ∑X_PUFAi_ ………………………… (8);

X_PUFAi_ = X_FAMEi_ * F_FAMEi-FAi_ ………………………… (9);

Note: X_Poly-UnsaturatedFat_ - poly-unsaturated fat acid content of sample, unit = g/100g;

X_PUFAi_ - single poly-unsaturated fat acid content in sample, unit = g/100g;

X_FAMEi_ - single poly-unsaturated fat acid methyl esters content in sample, unit = g/100g;

F_FAMEi-FAi_ - coefficient for fatty acid methyl esters were converted to fatty acids;

coefficient for fatty acid methyl esters were converted to fatty acids - F_FAMEi-FAi_ (S-Table 2).

Total-fat content of sample calculation was calculated according to formula (10);

X_TotalFat_ = ∑X_i_ * F_FAMEi-TGi_ ………………………… (10).

Note: X_TotalFat_ - total-fat content of sample, unit = g/100g;

X_i_ - single fat acid methyl esters content in sample, unit = g/100g;

F_FAMEi-TGi_ - coefficient for fatty acid methyl esters were converted to triglyceride (TG);

coefficient for fatty acid methyl esters were converted to fatty acids - F_FAMEi-TGi_ (S-Table 2).

Coefficient for fatty acid methyl esters were converted to triglyceride (TG) was calculated according to formula (11):

F_FAMEi - TGi_ = M_TGi_ * 13 M_FAMEi_ ………………………… (11).

Note: F_FAMEi_-T_Gi_ - coefficient for fatty acid methyl esters were converted to triglyceride (TG);

coefficient for fatty acid methyl esters were converted to fatty acids - F_FAMEi-TGi_ (S-Table 2);

M_TGi_ - fat acid triglyceride i molecular mass;

M_FAMEi_ - fat acid methyl esters i molecular mass.

The results preserved three significant digits.

**Table S5** Fatty acid methyl ester conversion factors

|  | FFAME-FA | FFAME-TG | FTG-FA |
| --- | --- | --- | --- |
| C11:0 | 0.93 | 0.9933 | 0.9363 |
| C14:0 | 0.9421 | 0.9945 | 0.9474 |
| C16:1 | 0.9477 | 0.995 | 0.9525 |
| C16:0 | 0.9481 | 0.995 | 0.9529 |
| C18:2n6c | 0.9524 | 0.9954 | 0.9567 |
| C18:1n9c | 0.9527 | 0.9955 | 0.957 |
| C18:0 | 0.953 | 0.9955 | 0.9573 |
| C20:4n6 | 0.956 | 0.09958 | 0.96 |
| C20:0 | 0.957 | 0.9959 | 0.961 |
| C22:6n3 | 0.959 | 0.9961 | 0.9628 |
| C22:1n9 | 0.9602 | 0.9962 | 0.9603 |

Note: FFAME-FA, the coefficient of converting fatty acid methyl esters to fatty acids; FFAME-TG, the coefficient of converting fatty acid methyl esters to single fatty acid triglycerides (1/3); FTG-FA, the coefficient of converting fatty acid triglycerides to fatty acids
